# Triage in the Time of Diphtheria

**DOI:** 10.5811/westjem.2020.6.46094

**Published:** 2020-08-21

**Authors:** Hadiki Habib, Hesty Kurniawaty

**Affiliations:** Emergency Unit, Dr. Cipto Mangunkusumo Hospital, Jakarta, Indonesia

## Abstract

**Introduction:**

A diphtheria outbreak occurred in 2017 in Jakarta, Indonesia, during which our hospital was appointed as a referral hospital where patients with upper respiratory tract symptoms were sent for confirmation of the diagnosis and medical intervention. In this study we review the implementation of the emergency department (ED) triage process and patient flow management during the diphtheria outbreak. No previous study in Indonesia has provided a detailed report on the triage process during infectious disease outbreaks.

**Method:**

We modified our pre-existing hospital triage method according to the “identify, isolate, and inform” principle. We developed novel criteria for triage to identify triage-suspected cases and also a diphtheria package to simplify the diagnostic process. Four separate rooms were modified to isolation spaces to enable medical staff to observe these patients. We obtained data from the ED outbreak registry and electronic health records.

**Results:**

Of 60 cases of triage-suspected diphtheria, six were classified as suspected diphtheria. The mean time from “identify” to “isolate” was 3.5 minutes, and from “isolate” to “inform” was 10 minutes. Mean ED length of stay for probable diphtheria was 24.46 hours. No medical personnel in the ED showed any signs of diphtheria 30 days after the outbreak had abated.

**Conclusion:**

The modified criteria can help triage officers detect suspected diphtheria cases and measure the triage response time. Use of the diphtheria package and four separate rooms in the ED could act as an infection control procedure and facilitate the improvement of the diagnostic process.

## INTRODUCTION

Diphtheria is a contagious infection caused by *Corynebacterium diphtheria.* The infection spreads through droplets and contact of mucous membranes with bodily fluid containing the causative bacteria. Diphtheria was last reported in 1987 and between 1990–1995 in the United Kingdom and the Soviet Union, respectively.^1^,^2^ Even though diphtheria can be prevented by immunization, it remains a major health problem in developing countries such as India, Thailand, and Bangladesh.^3^–^5^

During an outbreak, the emergency department (ED) has an important role in identifying suspected cases and providing acute medical management, while controlling infection transmission. It needs to act fast by implementing the World Health Organization (WHO) guidelines for suspected infectious disease.^6^,^7^

In 2017, a diphtheria outbreak occurred in Jakarta, Indonesia.^8^,^9^ Our hospital was appointed as a referral hospital where patients with upper respiratory tract symptoms were examined for case findings of suspected diphtheria.

Our goal in this study was to review the implementation of ED triage and patient flow management during the outbreak, as well as to present data on the new cases of diphtheria. No previous studies in Indonesia have reported on the triage process during infectious disease outbreaks.

## METHODS

### Participants, Study Design and Setting

We included all patients who presented to the ED with triage-suspected diphtheria. Informed consent was obtained, which included permission to take complete notes from medical records. This study was approved by the ethics committee of the hospital. We reviewed patients’ medical records from presentation to the ED until discharge. Data were collected during the outbreak in Jakarta from December 2017 to March 2018. We evaluated in-hospital transmission by interviewing medical personnel in the ED 30 days after the outbreak abated, to determine whether they showed signs of infection.

During the start of the outbreak, we vaccinated all medical personnel in the ED who were at risk of being exposed to diphtheria patients.

### Triage Criteria

We implemented the pre-existing, three-tier triage categories. Critical patients who required immediate treatment due to life-threatening conditions were included in the resuscitation category. In the urgent category we included non-critical patients whose symptoms were uncontrolled or could not be tolerated by the patient (eg, moderate to severe pain, vomiting, diarrhea, and severe dizziness) and who needed early medical intervention to prevent further clinical deterioration. Patients who did not belong to either of the two groups were classified into the non-urgent category.

### Diphtheria Diagnosis

Suspected diphtheria patients are defined as those with pharyngitis, laryngitis, or tonsillitis with greyish, thick pseudomembrane adhering to the pharynx, larynx, or mucosa of the nose that bleeds easily after applying light pressure using a wooden tongue depressor. Laboratory-confirmed diphtheria patients were defined as those with *Corynebacterium diphtheria* detected in their specimen cultures.^10^

### ED Outcome

We defined the outcome as the patient’s condition immediately before he or she left the ED. These were as follows: deceased; discharged as outpatient; transferred to inpatient ward; or referred to another hospital. The length of stay was defined as the time from admission to outcome determination in the ED.

### Identify, Isolate, and Inform Principle.^11^–^13^

To increase the awareness of the triage officer (nurse) on triage-suspected diphtheria, novel identifying criteria were included as follows: 1) those presenting with any one of the complaints such as sore throat, cough, or shortness of breath within less than three days of onset during the diphtheria outbreak; 2) those referred from other healthcare facilities with a diagnosis of suspected diphtheria; and 3) those who presented with a concern of having acquired the infection. The triage officer’s duty was to identify these conditions immediately after the patient arrived at the triage counter. Any one of the three criteria was sufficient to categorize the patient as having triage-suspected diphtheria and the diagnosis had to be clinically confirmed by an ear, nose and throat (ENT) specialist. A complete vital sign examination was performed after placing a surgical mask on the patient.^11^,^13^

**Population Health Research Capsule**

What do we already know about this issue?

Diphtheria is a re-emerging infectious disease. Proper triage and early recognition could improve emergency department flow and prevent local transmission.

What was the research question?

We sought to summarize our modification on of ED triage and patient flow during the diphtheria outbreak.

What was the major finding of the study?

Modified criteria help triage officers detect suspected cases and increase response time.

How does this improve population health?

This report highlights the importance of triage and early recognition to improve patient flow and prevent local transmission in the busy emergency department.

Triage-suspected patients were then transferred to a separate isolation room in the ED, to ensure that droplet precautions were done. There was no pressure gradient between the isolation room and the surrounding zone. Our unit provided four separate rooms for triage-suspected diphtheria, and each room could contain one patient. An emergency physician using a surgical mask received the patient in the isolation room and performed initial assessment and early intervention.

The “inform” process involved the emergency physician contacting the ENT specialist for throat examination by using the diphtheria package available in ED pharmacy. The diphtheria package consisted of one surgical mask with a face shield, a pair of non-sterile gloves, three cotton buds, one disposable apron, two disposable wooden tongue depressors, and a biohazard plastic bag.^13^

ENT specialist examination was then validated by an infectious disease specialist to designate the case as suspected diphtheria or not. A suspected case was reported by the hospital call center to the public health center and the infection center’s hospital for referral purposes.

### Data Management

We analyzed data descriptively, with no subgroup analysis. Incomplete filing in the medical records was considered as missing data.

## RESULTS

During the study period, 12,778 patients visited the ED of the Cipto Mangunkusumo Hospital. There were 60 cases of triage-suspected diphtheria, among which six were cases of suspected diphtheria. There were no initially non-suspected cases of diphtheria at our ED who later returned as suspected or diagnosed cases during the study period. Only suspected cases underwent microbiology culture.

The demographic characteristics of the patients with suspected diphtheria are shown in Table 1. Almost half of the triage-suspected cases (45%) were referred from outpatient primary care, paediatricians, and ENT specialist clinics. All referred patients presented to the ED on their own and brought the referral letter from the previous physicians. Upon arrival to the ED, the referred patient underwent a reassessment at the triage counter.

Among those with suspected diphtheria, five patients were brought by an ambulance to the infection center’s hospital to be admitted, and one patient was self-referred to our hospital. The mean time from “identify” to “isolate” was 3.5 minutes, and from “isolate” to “inform” was 10 minutes.

Patients with suspected diphtheria showed several observable signs and symptoms, as detailed in Table 2.

## DISCUSSION

Identifying suspected patients is an important protocol during an outbreak. Although not all patients with positive results during triage screening have the disease, deciding which patient group is at a high risk of contracting the disease and in need of further medical examination is an important part of infection control.^14^ Syndromic surveillance is the process of identifying symptoms and signs indicative of the disease during screening.^15^

During the recent outbreak of diphtheria, we modified our ED to create four separate rooms to contain suspected cases. This measure was adapted from that followed in Taiwan and Toronto during an epidemic of severe acute respiratory syndrome (SARS) where a temporary, high-efficient filtration system unit was built outside the ED for screening and isolating SARS patients.^7^

The “identify, isolate, and inform” method had been used previously during the Middle East respiratory syndrome coronavirus, Ebola virus disease, and measles epidemics.^7^,^11^,^12^,^16^ By using this method for triage, we were able to measure the response time (“identify” to “isolate” and “isolate” to “inform” time) for all suspected diphtheria cases that presented to the ED (Table 1). ED length of stay was prolonged (24.46 hours) because of the diagnostic process and referral communication at the infection center’s hospital, which took time.

Half of the suspected diphtheria patients were adults. This is supported by the findings of a systematic review of diphtheria in children and teenagers.^17^ Adult patients with a history of diphtheria immunization can get infected later in life because of a decrease in immunity from vaccination with age. Therefore, booster vaccines are needed, especially during an outbreak.^18^

Diphtheria is often difficult to distinguish from other upper respiratory tract infections as the symptoms are not very specific (Table 2). Nandi reported that the most common form of diphtheria is pharyngeal diphtheria, and 70% of patients have no immunization history.^19^ The most common symptoms are tonsillar exudate, sore throat, dyspnea, and fever.^19^ Clinical signs, such as a greyish pseudomembrane, are still very common and enabled easy identification of diphtheria at our center. However, a report from Brazil found that only 52% of diphtheria patients manifest that sign.^20^

## LIMITATIONS

This was a single-center study in a referral teaching hospital, and thus did not represent the incidence in the community. Additionally, incomplete data records could have resulted in potential bias. Another limitation was that a follow-up of microbial culture results from probable cases was not done as suspected diphtheria was used as a working diagnosis and is dependent on clinical assessment.

## CONCLUSION

Modified criteria help triage officers detect suspected diphtheria cases and increase the triage response time. The diphtheria package and the four separate rooms in the ED could act as an infection control procedure and facilitate the improvement of the diagnostic process. Further multicenter studies should be conducted for outbreaks other than diphtheria.

## Figures and Tables

**Table 1:** Demographic characteristics of patients with triage-suspected diphtheria.

Characteristics	N (%)
Gender	
Male	29 (48.3)
Female	31 (51.7)
Age (years)	
<18	25 (41.7)
≥18	35 (58.3)
ED Admission	
Self-referred	33 (55)
Referred from other medical facilities	27 (45)
Chief Complaint	
Sore throat	51 (85)
Fever	45 (75)
Shortness of breath	1 (1.7)
Clinical Manifestation	
Neck mass	11 (18.3)
Membrane in respiratory tract mucosa	38 (63.3)
Exposure History	
Positive	4 (6.7)
Negative	56 (93.3)
Diphtheria Diagnosis	
Suspected Diphtheria	6 (10)
Not Diphtheria	54 (90)
Length of Stay (LoS)	Mean
Identify to Isolate (minutes)	3.5
Isolate to inform (minutes)	10
Emergency unit LoS probable diphtheria (hours)	24.46

*ED*, emergency department.

**Table 2:** Clinical manifestations among patients with probable diphtheria.

Name	Gender	Age	GCS	HR	RR	SBP	Temp	Sore throat	Fever	Dyspnea	Pseudo-membrane	Comorbidity	Emergency unit outcome
1	F	34	15	90	18	130	37	+	+	−	+	−	Referred
2	F	15	15	100	16	110	37	+	+	−	+	−	Referred
3	F	3	15	100	24	110	36.5	+	+	−	+	−	Referred
4	M	7	15	110	30	88	36.7	+	−	−	+	−	In-patient
5	M	5	15	100	20	110	37	+	+	−	+	−	Referred
6	M	2	15	130	24	110	36.8	+	+	−	+	−	Referred

*GCS*, Glasgow Coma Scale score; *HR*, heart rate (beats per minute); *RR*, respiratory rate (breaths per minute); *SBP*, systolic blood pressure; *temp*, body temperature; *referred*, sent the patient to infection control hospital for further in-patient management.

## References

[1] Walters RF (1987). Diphtheria presenting in the accident and emergency department. Arch Emerg Med.

[2] Dittman S, Wharton M, Vitek C (2000). Successful control of epidemic diphtheria in the states of the former Union of Soviet Socialist Republics: lessons learned. J Infect Dis.

[3] Saikia L, Nath R, Saikia NJ (2010). A diphtheria outbreak in Assam, India. Se Asian J Trop Med.

[4] Pantukosit P, Arpornsuwan M, Sookananta K (2008). A diphtheria outbreak in Buri Ram, Thailand. Se Asian J Trop Med.

[5] Finger F, Funk S, White K (2019). Real-time analysis of the diphtheria outbreak in forcibly displaced Myanmar nationals in Bangladesh. BMC Med.

[6] Barajas G, Zembower T, Silkaitis Ch (2017). Triage form optimization: travel and infectious disease screening. Am J Infec Control.

[7] Department of Homeland Security (2003). Infectious disease outbreaks. Establishing separate triage and assessment facilities.

[8] Tosepu R, Gunawan J, Effendy DS (2018). The outbreak of diphtheria in Indonesia. Pan Afr Med J.

[9] Karyanti MR, Nelwan EJ, Assyidiqie IZ (2019). Diphtheria epidemiology in Indonesia during 2010–2017. Acta Med Indones.

[10] World Health Organization (2018). Diphtheria. WHO Vaccine-Preventable Surveillance Standard.

[11] Koenig KL (2015). Identify-Isolate-Inform: A modified tool for initial detection and management of Middle East respiratory syndrome patients in the emergency department. West J Emerg Med.

[12] Koenig KL, Alassaf W, Burns MJ (2015). Identify-isolate-inform: a tool for initial detection and management of measles patients in the emergency department. West J Emerg Med.

[13] The College of Emergency Medicine (2014). Ebola Guidance for Emergency Departments.

[14] Evans MI, Galen RS, Britt DW (2005). Principles of screening. Semin Perinatol.

[15] Hiller KM, Stoneking L, Min A (2013). Syndromic surveillance for Influenza in the emergency department: a systematic review. Plos One.

[16] Centers for Disease Control and Prevention (2016). Identify, isolate, inform: Emergency department evaluation and management for patients under investigation (PUIs) for Ebola virus disease (EVD).

[17] Murhekar M (2017). Epidemiology of diphtheria in India, 1996–2016: implications for prevention and control. Am J Trop Med Hyg.

[18] Tansel O, Ekuklu G, Eker A (2009). Community-based seroepidemiology of diphtheria and tetanus in Edirne, Turkey. Jpn J Infect Dis.

[19] Nandi R, De M, Browning S (2003). Diphtheria: the patch remains. J Laryngol Otol.

[20] Santos LS, Sant’anna LO, Ramos JN (2015). Diphtheria outbreak in Maranhao, Brazil: microbiological, clinical and epidemiological aspects. Epidemiol Infect.

